# Squamous Cancers and Precancers of the Vulva: Emerging Diagnostic, Prognostic and Predictive Biomarkers in Pathology

**DOI:** 10.3390/cancers18101518

**Published:** 2026-05-08

**Authors:** Somayah Alsolami, Jennifer Ji, Lynn Hoang

**Affiliations:** 1 Pathology and Laboratory Medicine, Vancouver General Hospital and University of British Columbia, Vancouver, BC V5Z 1M9, Canada; 2 Laboratory Medicine, Department of Pathology, King Abdulaziz University, Jeddah 21589, Saudi Arabia

**Keywords:** vulvar squamous cell carcinoma, vulvar intraepithelial neoplasia, tumor classification, p53, p16, human papillomavirus, immunohistochemistry, biomarkers

## Abstract

Vulvar squamous cell carcinoma (VSCC) and its precursor lesions are relatively rare malignancies of the gynecologic tract. In recent years, international organizations and pathologic reporting guidelines endorse the subdivision of VSCC into human papillomavirus (HPV)-associated and HPV-independent types. There is also growing evidence for the further separation of HPV-independent into p53 abnormal and p53 wild-type cancers. Although the diagnosis and subclassification of VSCC is often straightforward, using immunohistochemical markers such as p16 and p53 as surrogate markers for high-risk HPV infection and *TP53* mutation respectively, rare and unusual scenarios exist that can complicate VSCC classification. Herein we discuss these challenging scenarios in VSCC classification, as well as emerging VSCC prognostic biomarkers such as cyclin D1. In addition, the pathologic diagnosis of VSCC precursor lesions, particularly those of HPV-independent type, are frequently challenging to distinguish from benign conditions of the vulva. We discuss the recent literature describing the added diagnostic value of immunohistochemical biomarkers p53, CK17, CK13, SOX2, GATA3, GLUT1 and others, which may be particularly helpful when morphology is inconclusive. It is anticipated that with improved VSCC classification and precursor recognition, avenues for more tailored therapeutic strategies and earlier therapeutic intervention can be achieved.

## Simple Summary

Vulvar squamous cell carcinoma (VSCC) and its precursor lesions benefit from a growing repertoire of tissue-based immunohistochemical biomarkers to aid in diagnosis, tumor classification and prognostication. In current pathology guidelines, it is recommended that VSCC and their precursor lesions be divided into human papillomavirus (HPV)-associated and HPV-independent types. We discuss the use of p16, p53 and cyclin D1 in the subclassification and prognostication VSCC, as well as challenging interpretative scenarios. We also discuss the use of several new biomarkers (CK17, CK13, SOX2, GATA3 and GLUT1) in the diagnosis of the notoriously challenging group of squamous precursor lesions. Incorporating biomarkers into the evaluation of VSCC and its precursor lesions will enhance early detection, improve prognostic accuracy, and enable more individualized treatment approaches to be developed for this rare cancer type.

## 1. Introduction

Vulvar cancer is rare, with an age-standardized incidence rate of 0.83/100,000 globally [1]. It predominantly affects post-menopausal women, but the incidence in women younger than 60 years is rising [2]. Currently, there are no established screening protocols for vulvar cancer, alongside challenges in the pathologic recognition of early precancers, making early detection and timely intervention challenging [3]. Hence, there are growing efforts to improve early precancer detection through advocacy and the application of molecular and immunohistochemical testing in pathology [4]. Simultaneously, the approach to vulvar cancer continues to evolve, aligning with paradigm shifts towards personalized medicine, with increasing emphasis on integrating molecular markers for tumor classification, prognostication and tailored treatment strategies. Most notably, p16 and p53 immunohistochemistry (IHC) are now widely endorsed tissue biomarkers in pathology to distinguish human papillomavirus (HPV)-associated (HPVA) from HPV-independent (HPVI) vulvar squamous cell carcinomas (VSCC) and their precursors, and these molecular subtypes have emerged as clinically meaningful predictors of outcome [5,6,7,8]. In addition, ongoing research into targeted agents and immunologic biomarkers signals a shift toward more personalized treatment strategies based on tumor molecular profiles [9].

This review summarizes the current landscape of biomarkers in vulvar cancer, specifically squamous cell carcinoma and its precancers, and highlights their roles in diagnosis, prognostication and therapeutic decision making.

## 2. Etiology and Pathogenesis of Vulvar Squamous Cell Carcinoma

**Figure 1:**
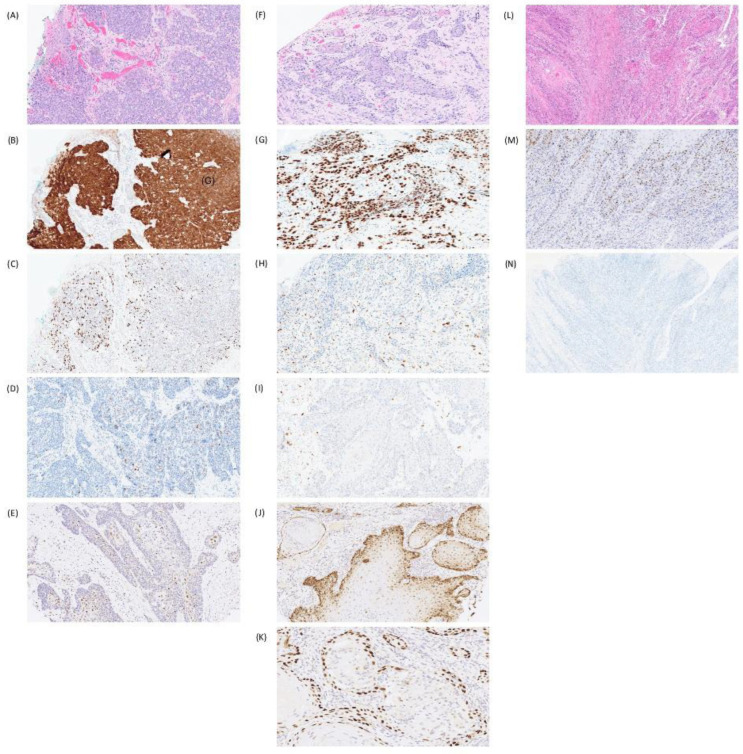
Vulvar squamous cell carcinoma (VSCC) subtypes. (**A**) VSCC, HPV-associated, where (**B**) p16 shows block-like cytoplasmic staining and (**C**) p53 shows a wild-type pattern. Examples of HPV-associated VSCC with (**D**) “null-like” staining pattern and (**E**) basal-staining pattern of p53. (**F**) VSCC, HPV-independent, showing (**G**) abnormal p53 in the form of parabasal/diffuse overexpression with (**H**) negative/weak staining of p16. Examples of HPV-independent VSCC with a (**I**) p53 null pattern of staining, (**J**) p53 cytoplasmic staining and (**K**) p53 basal-only overexpression. (**L**) VSCC, HPV-independent, with (**M**) p53 wild-type staining and (**N**) negative p16.

It is well-established that VSCC can occur through at least two biologic pathways, one which is driven by high-risk HPV infection (hrHPV) and the other occurring independently of HPV (Figure 1).

### 2.1. HPV-Associated Pathway

**Figure 2:**
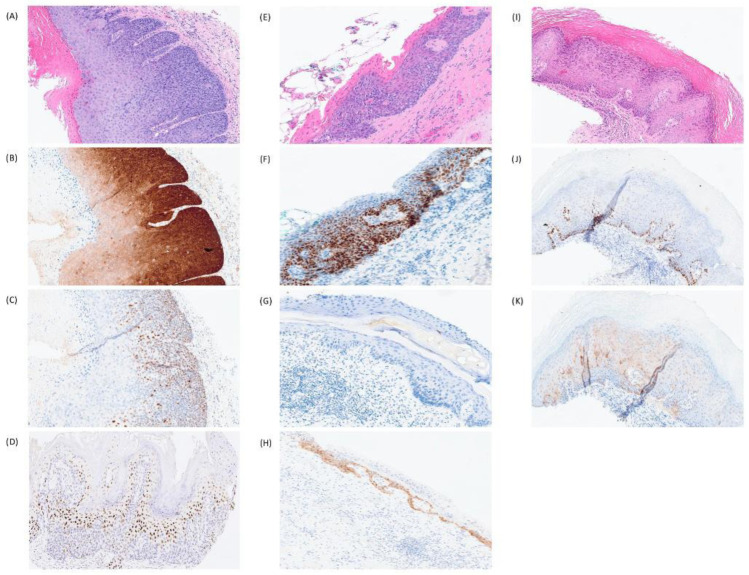
Vulvar intraepithelial neoplasia (VIN) subtypes. (**A**) High-grade squamous intraepithelial lesion (HSIL), HPV-associated, showing (**B**) p16 block-like staining and (**C**) p53 wild-type pattern. Example of (**D**) HSIL with basal-sparing p53 pattern. (**E**) Differentiated vulvar intraepithelial neoplasia (dVIN), HPV-independent, with (**F**) p53 abnormal parabasal/diffuse overexpression pattern. (**G**) An example of dVIN showing p53 abnormal null pattern and another case showing (**H**) p53 abnormal cytoplasmic pattern. (**I**) Verruciform acanthotic vulvar intraepithelial neoplasia (vaVIN), HPV-independent, showing (**J**) p53 wild-type staining pattern and (**K**) weak staining of p16.

HPVA VSCC generally occurs in younger women (~50 years of age), smokers, and immunosuppressed individuals [2]. This subtype typically arises from the precursor high-grade squamous intraepithelial lesion (HSIL), also known as vulvar intraepithelial neoplasia (VIN) of usual type (uVIN) (Figure 2) [9,10]. Oncogenesis in HPVA VSCC is primarily driven by hrHPV types, including 16, 18, 31, 33, 45, and 52 [11]. The HPV viral oncoproteins E6 and E7 play critical roles in oncogenesis; E6 degrades the tumor suppressor p53, and E7 degrades the retinoblastoma (Rb) protein leading to increased cell proliferation and causing the subsequent overexpression of p16 as a compensatory mechanism [12]. The overexpression of p16 IHC is frequently used as an indicator of hrHPV infection, as detailed below. In addition to cellular alterations caused by hrHPV, HPVA VSCC frequently harbor activating mutations in *PIK3CA*, members of the PI3K/AKT/mTOR pathway (*PTEN*, *STK11*, *FBXW7*, *SOX2*) and the chromatin remodeling gene *KMT2C* [13,14]. Although abrogation of p53 function occurs through HPV cellular mechanisms, mutations in *TP53* are rare [10,14].

### 2.2. HPV-Independent Pathway

In contrast, HPVI VSCC usually affects post-menopausal women, and is often associated with conditions such as lichen sclerosus (LS) and chronic inflammation [7,15,16]. The HPVI pathway is often characterized by *TP53* mutations, but a subset of HPVI tumors are *TP53* wild-type [17]. HPVI p53 abnormal (HPVI/p53abn) VSCC typically develop from differentiated vulvar intraepithelial neoplasia (dVIN; synonymous with HPVI/53abn VIN), while HPVI p53 wild-type (HPVI/p53wt) VSCC arise from p53 wild-type precursors, which have been assigned a variety of names, including vulvar acanthosis with altered differentiation (VAAD), differentiated exophytic vulvar intraepithelial lesion (deVIL), verruciform lichen simplex chronicus (LSC), verruciform acanthotic VIN (vaVIN) and vulvar aberrant maturation (VAM) (Figure 2) [16,18]. The incumbent World Health Organization Blue Book on Female Genital Tract tumors advocates for the use of HPVI p53wt VIN terminology, moving away from morphologic descriptors.

dVIN carries a higher risk of progression to VSCC and has a shorter interval to invasion compared to HSIL [19,20]. Aside from *TP53*, HPVI VSCC harbor frequent mutations in *CDKN2A*, *NOTCH1*, *FAT1* and *TERT* gene alterations [13,14,21,22].

## 3. Invasive Vulvar Squamous Cell Carcinoma

### 3.1. p16 as a Diagnostic, Prognostic and Predictive Biomarker

p16 IHC is a widely used and reliable surrogate marker for hrHPV infection in squamous (and glandular) lesions of the lower genital tract in pathology [23]. Strong and diffuse block-like cytoplasmic (and nuclear) positivity involving at least the lower one-third of the epithelial thickness is defined as p16-positive. This staining pattern is characteristic of HPVA VSCC and uVIN/HSIL. In contrast, HPVI VSCC and HPVI VIN demonstrate absent or only patchy p16 expression [10]. p16 IHC tends to also have higher sensitivity than HPV DNA- and RNA-based assays, which are limited in the number of HPV types tested, and thus the College of American Pathologists 2024 and International Collaboration on Cancer Reporting 2023 allow for p16 IHC as a surrogate marker for HPV status [5,8,24]. This is fortuitous as more pathology laboratories on a global scale will have p16 IHC accessible to them than direct HPV testing.

**Figure 3:**
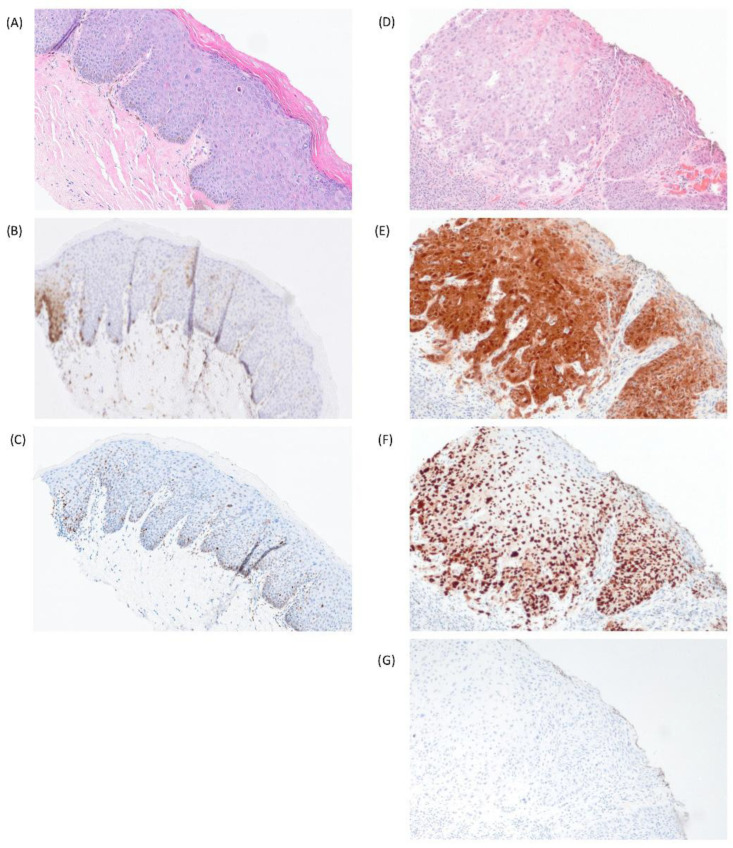
Cases with unusual/outlier staining patterns. (**A**) High-grade squamous intraepithelial lesion (HSIL), showing (**B**) unexpected negative (non-block) staining for p16 and (**C**) wild-type p53. The presence of high-risk HPV was confirmed by HPV RNA in situ hybridization. (**D**) Vulvar squamous cell carcinoma, HPV-independent, with adjacent vulvar intraepithelial neoplasia (VIN), showing (**E**) p16 diffuse strong block-like positivity but also (**F**) abnormal p53 parabasal-diffuse overexpression. (**G**) HPV RNA in situ hybridization was negative and a *TP53* mutation was identified by sequencing.

In rare cases, p16 may be false negative in <1% of HPVA VSCC (Figure 3) [10,25]. In cases where there are pathologic features to suggest HPV infection (e.g., warty or basaloid histology, koilocytic atypia, karryorhexis, background of morphologic HSIL), but p16 IHC is negative, testing for p53 IHC showing a basal-sparing pattern (a pattern specific to hrHPV infection) and/or HPV testing by RNA in situ hybridization (ISH) or DNA sequencing will be helpful [10,25,26]. Conversely, p16 IHC can be false positive in ~2% of HPVI VSCC [10,25]. This can be problematic when p53 IHC also exhibits an abnormal expression pattern—resulting in a so-called “double positive” p16/p53-positive tumor. In these cases, additional testing for HPV and *TP53* sequencing can be informative, if available. In the series by Yang et al., 5/225 showed a “double-positive” p16/p53 pattern (four overexpressed, one null). Subsequent testing for HPV by RNA ISH was negative and *TP53* sequencing showed mutations in all five cases, suggesting these “double-positive” p16/p53 cases would be best classified into the HPVI category. In a case study by Sisuashvili et al., a VSCC occurred in a 21-year-old where the VIN lesion had the appearance of HSIL. p16 was negative in the VIN lesion but positive in the invasive tumor. In a recurrence a year later, the tumor was negative for p16. p53 was abnormal in all components. HPV67 (low risk) was detected in the first tumor and HPV66 (high-risk) was detected in the recurrence, but HPV RNA ISH was negative in both instances. The authors found mutations in *TP53*, *CCND1* and *COL6A1*, more typical of HPVI VSCC, and suggested this tumor was best classified as an HPVI/p53abn, despite the patient’s young age. They propose the PCR result reflected environmental (not integrated) HPV (as the HPV ISH was negative) and that the p16 was false positive [27]. Overall, the number of “double-positive” p16/p53 VSCC in the published literature remains low [28,29], and further investigations using larger and pooled cohorts are needed to provide clarity to this uncommon scenario. In laboratories that lack access to ISH and *TP53* sequencing a classification of VSCC not otherwise specified (NOS) is acceptable.

Prognostically, p16 positivity is associated with more favorable clinical outcomes in VSCC. HPVA (p16-positive) VSCC typically demonstrates lower risk of locoregional recurrence and improved disease-free survival (DFS), progression-free survival (PFS), and overall survival (OS) compared with HPVI VSCC [30,31,32,33]. In the largest cohort study to date, of nearly 1300 cases, p16 positivity was a significant independent predictor of improved survival, particularly within advanced-stage disease, and this association persisted regardless of age or comorbidity [34]. These prognostic differences support the potential for treatment de-escalation in HPVA vulvar cancer, paralleling evolving management strategies in other HPV-driven malignancies such as oropharyngeal carcinoma [34]. There is also some evidence that HPVA VSCC may respond better to radiation than their HPVI counterparts, with lower rates of in-field relapse [35,36,37].

### 3.2. p53 as a Diagnostic and Prognostic Marker

There is growing interest in separating VSCC into three prognostic groups based on HPV and p53 status: HPVA, HPVI/p53wt and HPVI/p53abn. In general, HPVA tends to have the best prognosis, HPVI/p53wt bears an intermediate prognosis, and HPVI/p53abn VSCC has the worst clinical outcomes [17,21,33]. Kortekaas et al. reported a 5-year OS of 83%, 64% (hazard ratio [HR]: 2.16) and 48% (HR: 3.43) respectively in these three groups. A similar trend was observed for reference-free period [33]. Subsequent studies have found similar trends, except the study by Carreras-Dieguez et al. found the HPVI/p53wt group had the worst recurrence free survival (RFS) and intermediate disease-specific survival (DSS) amongst the three groups [38,39]. These differences are difficult to explain, but may be attributed to differences in radicality of surgical resection, re-excision rates and use of adjuvant therapy. Currently, there is no uniform consensus on the prognosis of HPVI/p53wt versus HPVI/p53abn groups—larger independent cohort studies will be valuable for shedding light on this issue.

Using p53 IHC, two wild-type patterns (scattered and mid-epithelial/basal-sparing) and four mutant patterns (basal overexpression, parabasal/diffuse overexpression, absent, and cytoplasmic expression) can be observed in VSCC and VIN [40,41]. Concordance between p53 IHC pattern and *TP53* mutation status is usually very high (>95%) [40], but lower concordance rates (~80%) have been reported [21]. Interpretation of p53 IHC in VSCC, is not always straight forward. The most common challenge has been to distinguish between strong wild-type staining and abnormal basal overexpression, which can represent approximately ~3–15% of cases [28,29,40]. Jeffus et al. assessed interobserver agreement in p53 (and p16) interpretation and found that interpretation of p53 (as wild-type vs. aberrant/abnormal) was only 50%, but did improve modestly to 70% after educational intervention [42]. In a study of 1293 VSCC and eight pathologists, concordance across all six p53 immunohistochemical patterns was 66.7%, and when binarized as p53 wild-type or mutant was 86.9% [25]. Similar to other sites in the gynecologic tract, optimization of p53 IHC protocols will also affect IHC interpretation [43].

Given the important role of p53 as a tumor suppressor, it is not surprising that p53 status would be an indicator of patient outcomes in VSCC. In a meta-analysis by Sand et al., abnormal p53 status was associated with worse OS (pooled HR: 1.81), but results varied by method of p53 analysis [44].

### 3.3. Cyclin D1 as a Prognostic Marker

Cyclin D1, encoded by the *CCND1* gene located on 11q13, is a cell cycle G1-S phase regulatory protein that is frequently amplified in many solid tumors, including head and neck squamous cell carcinoma [45], and its expression or gene amplification has emerged as a significant prognostic biomarker in VSCC, particularly in the HPVI subtype [21,46]. In the discovery study of 60 VSCC analyzed by whole exome sequencing, *CCND1* gains were found in one-third of VSCC and almost exclusively within the HPVI group. *TP53* mutation, *CCND1* gains and the combination of the two alterations were strongly associated with impaired RFS (HR 4.4; *p* < 0.001) and DSS (HR 6.1; *p* < 0.002). *CCND1* gains, or *ASH1L* mutations, were present in more than half of patients (65%) with recurrent disease and in almost all patients who died from disease (92%). p53 IHC status only maintained its prognostic significance, when combined with *CCND1* gains [21]. In a subsequent study by the same study group, the prognostic impact of cyclin-D1 IHC (*n* = 139 HPVI VSCC, overall cohort) and *CCND1* gains by sequencing (*n* = 54, sequencing cohort), and concordance between cyclin-D1 IHC and *CCND1* gain was evaluated. By using a 50% cutoff of tumor cells showing nuclear staining to define cyclin D1 overexpression, cyclin D1 IHC showed 94% sensitivity and 67% specificity for detecting *CCND1* gains. The presence of cyclin D1 overexpression was associated with advanced FIGO stage, lymph node metastasis, poor RFS and DSS. Strikingly, the mortality rate of *CCND1*-amplified tumors and cyclin-D1-overexpressed tumors was 55.6% and 28.8% respectively, compared to their normal counterparts with mortality rates of 8.3% and 4.5% respectively. Cyclin D1 overexpression and *CCND1* gains seemed to have a more pronounced impact on adverse prognosis than altered *TP53*/p53 in HPVI VSCC patients [46]. The prognostic significance of *CCND1* amplification and cyclin D1 in other studies has mixed results, likely stemming from varying definitions of cyclin D1 positivity, as there is no standardized cut-off in pathology practice [47,48,49].

### 3.4. Other Prognostic Markers for VSCC in Limited Studies

In a single study by Zannoni et al., the expression of ERα and ERβ was investigated in HPVI vulvar tumors to evaluate their changes during progression from normal epithelium to VSCC. ERα showed nuclear expression in normal epithelium and LS, whereas nuclear ERα expression was absent in dVIN adjacent to VSCC and in VSCC. They also observed a shift in ERβ expression from predominantly nuclear to cytoplasmic localization with malignant transformation. This cytoplasmic ERβ pattern was associated with tumor grade as well as shorter DFS and OS [50]. Several mediators of inflammation (COX-2, PPARγ, EP1 and EP4) have also been reported as prognostic markers in VSCC in a small number of studies [51,52,53].

## 4. Precursors to HPVI Vulvar Squamous Cell Carcinoma

**Figure 4:**
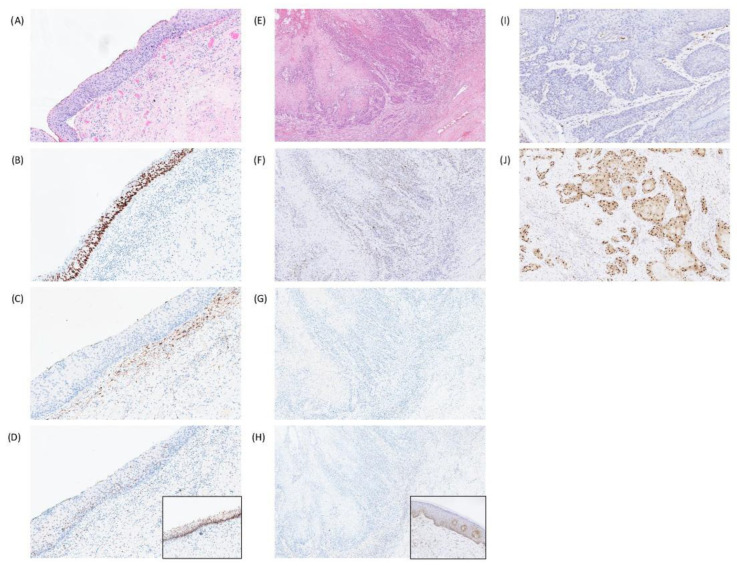
New diagnostic biomarkers for HPV-independent squamous neoplasia. (**A**) Differentiated vulvar intraepithelial neoplasia (dVIN) with (**B**) p53 parabasal/diffuse overexpression pattern, (**C**) p16 negativity and (**D**) loss of basal keratinocyte staining for GATA3. Inset shows normal GATA3 staining in adjacent squamous epithelium for comparison. (**E**) Vulvar squamous cell carcinoma, HPV-independent and (**F**) p53 wild-type, showing (**G**) complete loss of p16 staining with accompanying (**H**) loss of MTAP. Inset shows normal MTAP in adjacent squamous epithelium. (**I**,**J**) Examples of cyclin D1 staining from negative to diffuse/strong expression.

It is well known to practicing pathologists that diagnosing HPVI VIN lesions is challenging, due to their morphologic subtlety and overlap with a variety of non-neoplastic squamous lesions of the vulva. In contrast, the morphologic diagnosis of HPVA VIN (HSIL) is mostly straight-forward and we have therefore focused on reviewing pathologic biomarkers that aid in the diagnosis of HPVI VIN (Figure 4 and Table 1).

### 4.1. p53

Historically, the diagnosis of dVIN has been based on histologic assessment, and many studies have considered that dVIN can be p53wt or p53abn [22,55,56,61]. In more recent years, there has been a move to incorporate p53 into the definition of dVIN, whereby dVIN is synonymous with HPVI p53abn VIN [18]. This move is supported by a compelling study by Thuijs et al., showing that the 10-year cancer risk of HPVI p53wt VIN was 28% compared to 67% for HPVI p53abn VIN [19].

However, the interpretation of p53 IHC in VIN, as discussed above in the VSCC section, is fraught with challenges. Distinguishing between strong wild-type versus the abnormal basal overexpression pattern is especially challenging when proliferative or inflammatory non-neoplastic lesions are in the differential diagnosis (e.g., LS, lichen planus, LSC and spongiotic dermatitis) [55,56,73,74]. In an interobserver p53 IHC interpretation study in vulvar squamous (in situ) lesions, informed by *TP53* sequencing, the greatest challenge amongst pathologists was the distinction between the scattered and basal overexpression patterns, but this did improve from module A to module B after intervening feedback (*TP53* sequencing results) was provided (scores in the scattered pattern improved from 64.9% to 82.8%, +17.9%; scores in the basal overexpression pattern improved from 73.3% to 91.1%, +17.8%). Interpretation of p53 parabasal/diffuse overexpression patterns and absent/null patterns were not problematic, with accuracy rates >90% [74]. Given the wide and complex cellular roles of p53 in non-neoplastic and neoplastic processes, other biomarkers more specific for HPVI VIN are needed.

### 4.2. CK17

Aside from p53, CK17 has been the most studied IHC biomarker for the diagnosis of HPVI VIN, motivated by the literature documenting its usefulness in squamous dysplastic lesions across various mucocutaneous sites [55]. CK17 is an intermediate filament that is normally expressed in skin appendages (i.e., outer root sheath of hair follicles), and not in normal squamous epithelium. Multiple studies have found that CK17 is positive in the vast majority of HPVI VIN (89–100% of HPVI VIN diagnosed by morphology, 73–86% of HPVI p53abn VIN and 81–100% of HPVI p53wt VIN), with positive staining interpreted as moderate-to-strong suprabasal to full-thickness cytoplasmic expression [22,54,55,56,57,58,59,60,61]. This makes CK17 a highly sensitive marker that can assist in the diagnosis of these challenging cases. An absence of defined CK17 positivity would steer away from a diagnosis of HPVI VIN [56,59]. Importantly, the adjacent normal skin does not show abnormal CK17 expression and can be useful for comparison [54,56,58]. However, the findings are not always straightforward. A subset of these studies also reported CK17 positivity in non-dysplastic lesions such as LS and LSC, which may, under certain circumstances, be difficult to distinguish from HPVI p53wt VIN [22,54,56,58,59]. McMullen-Tabry et al. further found that dual staining for p53/CK17 is more diagnostically useful than either stain alone, and found a trend for higher frequency dual p53/CK17 expression in LS that progressed to dVIN [56]. Thuijs et al. also supported the dual use of CK17 and p53 for the diagnosis of HPVI VIN [59].

### 4.3. CK13

Dasgupta et al. acknowledged studies describing the progressive loss of CK13 with increased grades of squamous dysplasia in the oral cavity, cervix and esophagus. CK13 is normally expressed in the prickle cell layer of the normal squamous epithelium. The authors thus evaluated CK13 expression in dVIN and found decreased staining compared with LS and other non-dysplastic lesions. Complete loss of CK13, however, lacked sensitivity, as it was only seen in 8/54 (15%) of dVIN [55]. Similarly, a recent study by Altarawneh et al. found loss of CK13 in only 4/32 (12.5%) morphologic dVIN and 2/21 (9.5%) p53abn dVIN [61]. CK13 was not reliable as a standalone diagnostic marker and is most useful when interpreted together with CK17. The authors found that 8/21 (38.9%) p53abn dVIN showed full-thickness CK13 staining, the significance of which remains unclear [61].

### 4.4. SOX2

SOX2 is a transcription factor, regulator of pluripotent stem cells and is upregulated in a variety of squamous cell carcinomas in the body. SOX2 is expressed in normal squamous epithelium and LS [58,62], and shows increasing expression in VIN and VSCC [62]. Some studies have reported low sensitivity for the diagnosis of HPVI VIN [59,62], while others have reported high sensitivity [58,59], but a wide range of definitions for SOX2 positivity were used. Cook et al. found that SOX2 had additive value to CK17 and GATA3, in the diagnosis of vaVIN, where positivity for two or three markers was common in vaVIN (83%), providing a sensitivity of 83% and specificity of 88%. In contrast, most mimickers were positive for none or only one marker (85%) [58]. These observations emphasize the continued importance of histopathological assessment, supported by multiple markers, for accurate diagnosis.

### 4.5. GATA3

GATA3 regulates cellular differentiation and is another emerging biomarker for diagnosing challenging HPVI VIN. GATA3 is normally present in the basal layer and parabasal layers up to at least the mid stratum spinosum [64]. In the first study of GATA3 in VIN, GATA3 was abnormally lost (>25% loss of staining in the basal layer) in the majority of dVIN, with no loss in HSIL, and loss in the majority of VSCC (of both HPVA and HPVI types). Non-dysplastic epithelial lesions retained staining similar to normal skin [63]. Since then, others have reported GATA3 loss of staining in a small subset of HSIL and non-VIN mimickers (i.e., LS and verruciform xanthoma), but the overall sensitivity and specificity for GATA3 in the detection of dVIN remains high [58,64]. The sensitivity appears to be lower when applied to vaVIN [58]. Both Zare et al. and Cook et al. suggest using GATA3 in combination with other biomarkers (e.g., p53, p16, CK17, SOX2) to enhance diagnostic accuracy and mitigate its limitations in difficult cases [58,64].

### 4.6. GLUT1

GLUT1 is an indicator of hypoxia and is increased in a variety of malignancies as metabolism shifts from aerobic to anaerobic (Warburg effect). Motivated by a study on GLUT1 and VSCC from Mayer et al. [75], Zhang et al. recently studied GLUT1 in squamous lesions of the vulva and found that GLUT1 was overexpressed in the vast majority of HSIL, dVIN and VSCC. In addition, two distinct patterns of GLUT1 were observed—HPVA VIN and VSCC demonstrated overexpression of GLUT1 in the upper and intermediate layers (with negative or weak staining in the basal and parabasal layers), while HPVI VIN and VSCC demonstrated the opposite, staining mainly in the basal and suprabasal layers. The diagnostic utility of GLUT1 appears so far excellent, as it was overexpressed in both HPVI p53abn and p53wt VIN [65]. In a follow-up study, 24 vaVINs (eight associated with VSCC) all confirmed to be p53wt, showed the same pattern of GLUT1 overexpression and this was not observed in the 48 non-dysplastic epithelial lesions. The authors caution that the intensified peri-papillae pattern seen in the non-dysplastic lesions can be challenging to interpret, and advise that GLUT1 staining is typically around dermal papillae with or without focal suprabasal extension and there is often a strong-to-weak gradient from the peri-papillae basal to suprabasal layers [66].

So far, there is no clear indication which IHC biomarker (p53, CK17, CK13, SOX2, GATA3 or GLUT1) is superior to the rest. Their ancillary use will depend on their availability in pathology laboratories and experience of the practicing pathologist. Serum biomarkers are not well described for vulvar cancers.

### 4.7. Other Diagnostic Markers for HPVI VIN in Limited Studies

Other adjunct biomarkers to aid in the diagnosis of HPVI VIN have been in the form of single case studies. The value of survivin, hTERT, γ-H2AX, phosphorylated S6, ORF1p, CK5 and ProEx C is summarized in Table 1. In the oral cavity, where similar problems in diagnosing HPVI squamous dysplasia exist, there is some data to suggest that MTAP may be a helpful adjunctive marker in rare situations. In their small series, Ji et al. identified 19 cases of questionable oral squamous dysplasia. In five of 19 cases, p16 IHC demonstrated an unusual ‘null’ (completely negative) IHC pattern. The authors postulated that this may be due to homozygous codeletion of *CDKN2A* (which encodes p16) and its neighboring gene *MTAP*, a phenomenon which has been reported in mesotheliomas [76]. Four of the five cases with “null” p16 staining were found to have *CDKN2A* loss (homozygous deletion, heterozygous deletion, or monosomy for the *CDKN2A* locus) by fluorescence ISH (FISH), and all four cases show loss of MTAP on IHC [77]. The authors therefore suggest that MTAP IHC can be useful, when squamous dysplasia is in question and p16 is completely negative. In our pathology practice, we have observed rare cases of HPVI/p53wt VIN that have demonstrated immunohistochemical loss of MTAP (unpublished observations).

A group from Amsterdam has assessed the prognostic significance of DNA methylation in precursor lesions and the associated risk of progression to cancer. Using a 12 DNA methylation panel applied to VSCC, VIN adjacent to VSCC, VIN without VSCC and normal skin, the authors found increased methylation with disease severity [78]. In a large follow-up study using the same 12 DNA methylation markers in 751 VIN and 113 controls, SST was found to be the best performing individual biomarker, detecting 80% of overall high-grade VIN and 95% of HPVI VIN (95%) [79]. When the panel was narrowed down to a three-gene DNA methylation panel (ZNF582, SST, miR124-2), the presence of DNA methylation was a significant predictor for cancer progression in HSIL, but was not in dVIN, where p53 was the sole prognostic risk factor for progression to cancer [80]. This three-gene DNA methylation panel was also applied to LS, where the majority of LS cases that had VSCC had high methylation results together with p53 abnormalities [81].

## 5. Summary

The landscape of biomarkers used in pathology for the assessment of vulvar cancer has evolved significantly. p16 and p53 IHC are now essential for classifying tumors into prognostically distinct HPVA and HPVI subtypes. Beyond classification, emerging biomarkers offer potential for risk stratification and personalized management options. Diagnostic challenges, particularly in identifying HPVI precursor lesions, are being addressed with adjunctive IHC markers including CK17, CK13, SOX2, GATA3 and GLUT1. As our molecular understanding deepens, integrating these biomarkers into clinical practice will be crucial for improving early detection, refining prognostic assessment, and guiding targeted therapy in vulvar cancer.

## Figures and Tables

**Table 1:** Emerging diagnostic biomarkers for HPV-independent vulvar intraepithelial neoplasia.

Biomarkers	Number of Studies	Interpretation Criteria	Total Number of Cases (and % Positive) by Interpretation Criteria	Sensitivity †	Specificity †
CK17 Summary	9	Positive expression is Diffuse, moderate to strong cytoplasmic staining, suprabasal or full-thickness expression.	300 VIN 217 non-dysplastic lesions including LS	Relatively high sensitivity (70–100%)	Wide range of specificity (27–100%)
Podoll 2016 [54]		intermediate to strong diffuse immunoreactivity, confined to the upper half of the epithelium	29 (93%) dVIN 9 (0%) HSIL 7 (29%) LSC 8 (63%) LS	93%	53%
Dasgupta 2018 [55]		diffuse, strong staining, suprabasal to full thickness	54 (88.9%) dVIN 14 (0%) LS 30 (0%) NDEL	89%	100%
Dasgupta 2021 [22]		diffuse, moderate-strong, cytoplasmic staining, suprabasal to full thickness	56 (80%) dVIN 8 (88%) deVIL 27 (63%) HSIL 46 (20%) NDEL	80% for diagnosis of DVIN (by ROC)	80% for diagnosis of DVIN (by ROC)
McMullen-Tabry 2022 [56]		moderate to strong, partial thickness or full thickness staining	30 (100%) dVIN 30 (73%) NDEL (LS, LSC, PEH) 20 (75%) LS progressed to dVIN 20 (50%) LS did not progress to dVIN 5 (0%) normal	100%	27%
Hartsough 2024 [57]		superficial to suprabasal expression	10 (70%) vaVIN (vLSC, DE-VIL, VAAD)	70%	n/a
Cook 2024 [58]		Full thickness	16 (81%) HPVI p53wt VIN (vaVINs) 34 (32%) NDEL (verruciform xanthoma, LSC *, LS *, psorasis, PEH *)	81%	68%
Thuijs 2024 [59]		diffuse (>50%) and moderate-to-strong intensity, partial to full thickness	30 (73%) HPVI p53abn VIN 16 (100%) HPVI p53wt VIN 58 (14%) HSIL 4 (0%) LSIL 37 (24%) NDEL 5 nonconclusive	83%	75%
Hartsough 2025 [60]		Scored from 0 to 3 based on intensity and thickness (superficial, suprabasal, diffuse)	11 VSCC HPVI 19 dVIN 8 atypical LS 11 LS (increasing scores with progression)	n/a	n/a
Altarawneh 2026 [61]		Full thickness	32 (90.6%) dVIN [85.7% if p53abn dVIN]	91%	n/a
CK13 Summary	2	Reduced expression	86 dVIN 44 non-dysplastic	Low sensitivity ~15%	Uncertain
Altarawneh 2026 [61]		either lack of staining or diffuse full-thickness	Lack of staining: 32 (12.5%) dVIN [9.5% of p53abn dVIN] Diffuse full thickness: 32 (46.9%) dVIN [38% of p53abn dVIN]	n/a	n/a
Dasgupta 2018 [55]		complete lack of staining	54 (15%) dVIN 14 (0%) LS 30 (0%) NDEL	15%	100%
SOX2 Summary	4	Diffuse moderate to strong nuclear staining	140 HPVI VIN 122 non-dysplastic lesions	Wide range 33–86%	Wide range 20 to 100%
Brustmann 2013 [62]		modified score combining proportion and intensity divided into negative, moderately positive (score 3–4) and strongly positive (score 5–6)	33 (100%) SCC 18 (100%) dVIN 16 (100%) HSIL 9 (56%) LS 25 (88%) normal (using score of 3–4)	44–100% (depending on cut-off of score 3–4 or 5–6)	20–100% (depending on cut-off of score 3–4 or 5–6)
Dasgupta 2021 [22]		diffuse, moderate to strong nuclear expression, in basal/suprabasal layers to full thickness	56 (86%) dVIN 8 (88%) deVIL 27 (88%) HSIL 46 (19%) NDEL	Sensitivity 86% for diagnosis of DVIN (by ROC)	specificity 81% for diagnosis of DVIN (by ROC)
Cook 2024 [58]		strong staining ≥ 10% cells	12 (75%) vaVINs 30 (17%) NDEL (verruciform xanthoma, LSC, LS, psorasis, PEH)	75%	83%
Thuijs 2024 [59]		diffuse >50% and moderate-to-strong intensity, partial to full thickness	30 (43%) HPVI p53abn VIN 16 (13%) HPVI p53wt VIN 58 (2%) HSIL 4 (0%) LSIL 37 (3%) NDEL (LS, inflammation, reactive, fibroepithelial polyps, normal)	33%	97%
GATA3 Summary	3	Positive is loss of staining in >25% of basal layer	77 HPVI VIN 113 non-dysplastic lesions	~90% for dVIN 58–90% for vaVIN	~95% for dVIN 78–90% for vaVIN
Goyal 2018 [63]		loss in >25% of basal cells	23 (87%) VSCC 34 (88%) dVIN 30 (0%) HSIL 20 (0%) LS 12 (0%) LSC 45 (0%) normal	88%	100%
Zare 2023 [64]		loss in >25% of basal cells	21 (90%) dVIN 10 (90%) VAM 44 (16%) HSIL 49 (4%) NDEL (* LS, LSC, SD, LP, inflammation) 75 (0%) normal	90%	96%
Cook 2024 [58]		loss in >25% of basal cells	12 (58%) vaVINs 32 (22%) NDEL (verruciform xanthoma *, LSC, LS, psorasis, PEH)	58%	78%
GLUT1	2	Moderate to strong diffuse staining in basal to mid-epithelial layers	89 HPVI VIN 88 non-dysplastic lesions	Very high sensitivity reported so far	Very high specificity reported so far
Zhang 2025a [65]		Moderate to strong staining compared to nonneoplastic vulva	90 VSCC (88.9–100%) 65 (96.9%) HPVI VIN [98% of p53abn and 92% of p53wt] 45 (82.2%) HSIL 40 (0%) NDEL (LS, inflammation, FEP, other)	97%	100%
Zhang 2025b [66]		diffuse confluent staining with moderate to strong intensity, in basal to intermediate cell layers	24 (100%) vaVIN (all p53wt) 48 (0%) NDEL (resection margins, inflammation, LSC, seborrheic keratosis, PEH, condyloma)	100%	100%
Survivin	2	Moderate to strong nuclear over-expression in >5% of cells	52 VSCC 16–34 HPVI VIN ‡ 20 non-dysplastic lesions, including LS ‡	High sensitivity depending on cut-off used to define positivity	Uncertain (not many non-dysplastic epithelial lesions studied)
Brustmann 2011 [67]		Moderate to strong nuclear over-expression in >5% of cells	20 (100%) VSCC 16 (87%) dVIN 16 (100%) HSIL 10 (40%) LS 25 (20%) normal	87%	n/a
Wellenhofer 2012 [68]		>5% nuclear immunoreactive cells (score of 2+ or 3+)	32 (100%) VSCC 18 (100%) dVIN 16 (100%) HSIL 10 (40%) LS 25 (20%) normal	100%	n/a
γ-H2AX Brustmann 2011 [67]	1	Moderate to strong nuclear over-expression in >5% of cells	20 (40%) VSCC 16 (69%) dVIN 16 (69%) HSIL 10 (30%) LS 25 (0%) normal	69%	n/a
hTERT Wellenhofer 2012 [68]	1	>5% nuclear immunoreactive cells (score of 2+ or 3+)	32 (100%) VSCC 18 (100%) dVIN 16 (100%) HSIL 10 (40%) LS 25 (0%) normal	100%	n/a
P-S6 Pinto 2013 [69]	1	Positive basal-layer staining	7 (100%) dVIN 9 (77%) HSIL	n/a	n/a
ORF1p Hofstetter 2023 [70]	1	Moderate-to-strong basal or full-thickness staining	29 (93%) dVIN 26 (77%) HSIL 20 (30%) inflammatory 22 LS (18%) 20 (0%) normal	93%	76%
CK5 Zhang 2016 [71]	1	Reduced/decreased staining in VSCC compared to healthy epithelium and DVIN (mass spectrometry and later IHC)	6–8 VSCC with dVIN and normal skin	n/a	n/a
ProEx C (MCM2/TOP2A) Chen 2010 [72]	1	Nuclear overexpression extends beyond basal/parabasal layer	18 VSCC 3 verrucous carcinoma 6 dVIN 23 HSIL 14 condyloma 13 LS 22 normal	0%	n/a

* Pertains specifically to, † in reference to HPVI VIN only, ‡ unclear if independent or overlapping cases between two studies. Abbreviations: deVIL: differentiated exophytic vulvar intraepithelial lesion; dVIN: differentiated vulvar intraepithelial lesion; HPV: human papillomavirus; HPVI: HPV-independent; HSIL: high-grade squamous intraepithelial lesion; LP: lichen planus; LS: lichen sclerosus; LSC: lichen simplex chronicus; LSIL: low-grade squamous intraepithelial lesion; n/a: not applicable; NDEL: non-dysplastic epithelial lesions; p53abn: p53 abnormal; p53wt: p53 wild-type; PEH: pseudoepitheliomatous hyperplasia; ROC: receiver operating characteristic; SD: spongiotic dermatitis; VAM: vulvar aberrant maturation.

## Data Availability

No new data was generated for this review paper.
